# Ultrasound-Guided Pulsed Radiofrequency Treatment for Superficial Peroneal Nerve Entrapment in a Professional Handball Player

**DOI:** 10.7759/cureus.42043

**Published:** 2023-07-17

**Authors:** Rui Martins da Silva, Adriana Pereira, Raquel Branco, José Luís Carvalho

**Affiliations:** 1 Sports Medicine, Futebol Clube do Porto, Porto, PRT; 2 Physical Medicine and Rehabilitation, Centro de Medicina de Reabilitação de Alcoitão, Cascais, PRT; 3 Physical Medicine and Rehabilitation, Hospital do Divino Espírito Santo, Ponta Delgada, PRT; 4 Physical Medicine and Rehabilitation, Centro de Reabilitação do Norte - CHVNG/E, Vila Nova de Gaia, PRT; 5 Sports Medicine, Federação Portuguesa de Futebol, Lisboa, PRT

**Keywords:** pain, pulsed radiofrequency, ultrasound, athlete, entrapment, peripheral neuropathy, superficial peroneal nerve

## Abstract

Peripheral nerve injury in athletes is rare but deleterious to their performance, entrapment being one of the most frequent causes. Isolated injury to the superficial peroneal nerve (SPN) is rare and often underdiagnosed. The authors reported a clinical case of a 34-year-old handball athlete who presented with neuropathic pain in the inferolateral third of the leg and dorsum of the foot, with three months of evolution, after an ankle sprain, refractory to conservative treatment. After clinical assessment and ultrasound investigation, it was considered that the pain source was likely to be an SPN entrapment. Thus, a diagnostic ultrasound-guided nerve block with 2ml of 2% lidocaine and 3ml of 0.2% ropivacaine was performed, followed by nerve hydrodissection, with a major improvement in the patient's symptoms and functionality for three weeks. Thereafter, a long-lasting alternative was made - pulsed radiofrequency (pRF). There were no complications after the procedure. As a form of neuromodulation, pRF offered pain resolution without tissue damage or painful sequela, after 3, 6, 12, and 24 months of follow-up and complete participation in sports activity, avoiding surgical treatment. With this clinical case, the authors intend to demonstrate the effectiveness of pRF in the resolution of peripheral neuropathy due to entrapment, avoiding more invasive treatment options and, in the case of an athlete, allowing an early return to play. They also intend to corroborate the advantage of using ultrasound in the diagnosis and guide of minimally invasive procedures.

## Introduction

Peripheral nerve injury in athletes is a rare condition but has a negative impact on performance and playing ability [1-2]. Peripheral neuropathy (PN) secondary to entrapment is a common cause, with peroneal neuropathy being the most common lower limb mononeuropathy seen in athletes [1-2]. Isolated superficial peroneal nerve (SPN) abnormalities are rarely present and are mostly underdiagnosed [2-3]. Studies report an incidence of SPN neuropathy of 3.5% in patients with chronic leg pain [2].

SPN originates from the common peroneal nerve near the fibular neck in the proximal leg, runs through the intermuscular septum between the anterior and lateral compartments of the leg (between the fibularis brevis, fibularis longus, and extensor digitorum longus muscles), and pierces the crural fascia to exit the lateral compartment into the subcutaneous tissue of the lower third of the leg. After providing muscular innervation to the fibularis longus and fibularis brevis muscles, it descends to the foot where it divides into two sensory terminal branches: medial and intermediate dorsal cutaneous nerves, which supply sensation to the lower third of the anterolateral leg and to the dorsum of the foot and toes, except in the first web space and lateral and medial borders of the foot [3-5].

Several causes of SNP entrapment have been described. The most common are ankle inversion sprains, direct trauma to the anterolateral aspect of the leg, fibula fractures, surgical sequelae, and overstretching of the anterolateral leg [2-4, 6]. Soccer, hockey, football, and dancing are the sports most often involved [2-4, 6-7]. Furthermore, entrapment can develop after injury due to adhesions secondary to chronic inflammation, edema, hematoma, infection, cast immobilization, and connective tissue disease [1, 3-4, 6-7].

Patients with entrapment of SPN usually present pain in the distal anterolateral leg and dorsum of the foot sparing the first web space, which can assume neuropathic characteristics. This may be associated with sensory changes (paresthesia, hypoesthesia, hyperesthesia). In the early stages, pain and sensory symptoms worsen with exercise and are the main complaints; later, there may be a decrease in the strength of foot eversion. Tinel's sign is usually positive [1-5].

An accurate and prompt diagnosis is of utmost importance for a better therapeutic approach and to prevent irreversible nerve damage [1, 3, 6]. Although the diagnosis is essentially clinical, electrodiagnostic studies (EMG) can help and can determine the type, location, severity, and prognosis of the lesion [8]. Radiography (X-ray) can exclude fractures and bone tumors and can diagnose any misalignment or instability that may be causing the pain [2-3, 7]. Ultrasound (US) and magnetic resonance imaging (MRI) allows us to follow the trajectory of the nerve and to identify areas of hypertrophy, fascicle heterogeneity, edema, and static or dynamic compression [4, 9-10]. MRI has been shown to have variable accuracy. On the other hand, US enables a dynamic assessment and has become one of the preferred modalities for peripheral nerve examination, diagnosis, and guidance of interventions [9-11]; this dynamic image can also give us a correlation with the clinic [10].

SPN is easily seen by US between the peroneus longus and extensor digitorum longus muscles, at the intramuscular septum between the anterior and lateral compartments of the leg and traversing the crural fascia [3-4, 9-10].

Differential diagnoses of persistent/chronic anterolateral leg and ankle pain include chronic ankle instability (which includes the superior and inferior syndesmosis instability), bone bruises, stress fractures, chondral injuries, sinus tarsi syndrome, tendon pathology of the dorsiflexors and evertors of the foot, common peroneal or deep peroneal neuropathy, lateral leg compartment entrapment syndrome, or L5 radiculopathy [1-2, 5, 7].

The initial treatment for this entrapment neuropathy is conservative, including physiotherapy, correction of biomechanical defects, and pharmacological treatment with nonsteroidal anti-inflammatory (NSAIDs), analgesics, tricyclic antidepressants, serotonin norepinephrine reuptake inhibitors, gabapentinoids, and vitamin B1- and B12-based medication [1-2, 6, 12-13]. If symptoms persist after three to six months, surgical release and neurolysis must be considered, with 50-85% of success [1, 3, 12]. Recently, some other less invasive therapeutic options have been described, namely, nerve hydrodissection, nerve blocks, and ablative/continuous or pulsed radiofrequency (pRF) [3, 9, 11-13]. Non-ablative pulsed pRF neuromodulation is an interventional pain management method. In contrast with continuous radiofrequency, pRF offers the advantage of pain control without tissue destruction or painful sequelae. This is especially alluring in neuropathic pain [14-16]. In this case report, we describe the successful management of isolated SPN entrapment neuropathy, in a professional handball player, with ultrasound-guided pRF of the SPN.

## Case presentation

This case report refers to a 34-year-old male who is an elite professional handball player. His personal history was unremarkable (except for a left tibia and fibula fracture (2011)) and underwent conservative treatment (returning to play after eight months) and a surgical reconstruction of the left anterior cruciate ligament (ACL) with semitendinosus-gracillis graft (2017), followed by a rehabilitation plan, returning to play after nine months. In January 2021, he presented to the outpatient department of Physical and Rehabilitation Medicine with pain in the inferolateral third of the left leg radiating to the dorsal aspect of the foot, with three months of evolution. He suffered a left ankle sprain (grade II) four months earlier and underwent conservative treatment, returning to training after 25 days. The radiating pain and burning sensation were worse during exercise, especially with lateral displacements and landings. Deep friction/massage techniques of conventional treatment on the anterior surface of the leg were also painful. Oral analgesics, NSAIDs, muscle relaxants, pregabalin, and lidocaine patch were not effective. Consequently, he missed 75% of team practice and more than 50% of games during this period. He scored a maximum pain intensity of 8-9/10 in training and 3/10 at rest, on the numeric pain scale (NPS).

On examination, there was no visible ankle edema, and active and passive knee and ankle range of motion were preserved. Knee instability was excluded, and ankle instability tests were all negative (anterior and posterior drawer, eversion and inversion stress, talar tilt, and squeeze test). Neurological examination was unremarkable, including muscle strength, deep tendon reflexes, and superficial sensation test. The pain was not elicited on palpation of the inferior-anterior tibioperoneal ligament, interosseous membrane, and proximal and distal tibiofibular joints. He had muscle tension in the anterolateral aspect of the left leg and a dubious Tinel's sign of the SPN as it crosses the crural fascia.

An imaging evaluation was carried out. Leg, ankle, and foot X-rays were normal. The computed tomography (CT) scan of the leg, ankle, and foot showed sequelae of an old tibia and fibula fracture, associated with a slight calcification of the distal syndesmosis. MRI excluded effusion, bone or soft tissue swelling, condral, subcondral, or bone marrow changes and prominent ligamentous changes on the ankle. Surgery was proposed by an orthopedic surgeon to stabilize the distal tibioperoneal syndesmosis.

**Figure 1:**
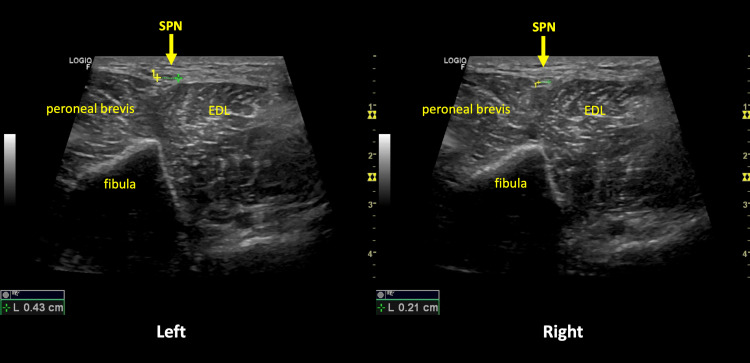
Superficial peroneal neuroma - differences between the left and right sides. SPN - Superficial peroneal nerve. EDL - Extensor digitorum longus.

The authors performed a US scan with a 3.7-13 MHz linear probe (LogiqTM P8 ultrasound machine, GE Healthcare, Chicago, Illinois, United States), which revealed an area of hyperechogenic nerve thickening, with fascicular nerve heterogeneity, asymmetric in relation to the contralateral at the same level (Figure 1), compatible with an SPN neuroma, most likely because of entrapment or stretching. This was associated with a doubtful Tinel's sign in the same location (13cm from the lateral malleolus), between the peroneal brevis muscle and the extensor digitorum longus muscle.

A diagnostic test with a US-guided nerve block with a short-acting anesthetic was made. For the procedure, the patient was positioned supine, and the SPN was tracked with US starting from the common peroneal trunk at the fibular neck, checking for changes in the normal morphology and characteristics of the nerve, until the thickened area corresponding to the neuroma/entrapment was found. After the aseptic measures, a 22-gauge needle (0.8x40mm) was guided in a lateral to medial approach to the target, and a mixture of 2ml of 2% lidocaine and 3ml of 0.2% ropivacaine was injected around the nerve. After five minutes the patient became pain-free, even in exercises with lateral displacements and landings, and the diagnostic test was considered positive.

Three days and two team training sessions after, he referred a 70-80% symptom relief. Therefore, it was decided to do a second SPN US-guided block, associated 0.5ml of 40mg/ml methylprednisolone, and a peripheral nerve hydrodissection with 10ml of 0.9% saline solution (Video 1 and Video 2). This resulted in total pain relief, and the athlete was able to participate in all team training sessions and in five matches.

**Video 1:** Superficial peroneal nerve block and hydrodissection. SPN - Superficial peroneal nerve. EDL - Extensor digitorum longus.

**Video 2:** Final result of the superficial peroneal nerve block and hydrodissection. SPN - Superficial peroneal nerve. HD - hydrodissection. EDL - Extensor digitorum longus

Three weeks later, he reported a 50% worsening of the complaints (with NPS 5/10 during sports practices and 2/10 in rest), whereby a more prolonged alternative was considered - pRF at the entrapment site. Since the SPN is a mixed nerve and provides cutaneous innervation, the use of continuous RF is not recommended because of the high risk of injury of the motor fibers and of sensory changes (paresthesia, dysesthesia, and hypoesthesia) [15-16]. Therefore, in this case, it was preferred for the neuromodulation effect of pRF, with no nerve injury associated. The US-guided procedure was performed using a specific 22-gauge radiofrequency cannula - 10cmx10mm (active tip)x22ga (0.7mm) - with the tip positioned perpendicular to the nerve (nerve short axis). Sensitive and motor tests were performed for additional confirmation, and in this case and at this level, there was eversion and visible contraction on ultrasound only of the peroneal brevis and paresthesia to the dorsum of the foot and toes, except in the first web space. The pRF was conducted with the following parameters: 100V, frequency of 2Hz, and 42ºC, with pulses of 2ms, for three minutes. After the pRF cycle, 3ml of 0.2% ropivacaine was administered around the nerve. There were no complications during or after the procedure. The patient presented an immediate response after the local anesthetic administration, scoring his pain as 0/10 (NPS).

This procedure allowed the athlete's return to play two days later, without complaints or secondary effects. At 3-, 6-, 12- and 24-month follow-ups, the athlete still had no pain or functional limitations, no secondary effects or complications were seen, and he was able to perform all the team's training sessions and games.

## Discussion

Peripheral neuropathies are challenging to identify and may be underdiagnosed. Therefore, they should be more frequently considered, especially when pain has neuropathic features such as the radiation and burning sensation in this case, associated with a previous injury that can be pathophysiological or biomechanically related (ankle sprain). It should also be suspected when the symptoms are refractory to conventional treatment (pharmacological and physiatric treatment).

The diagnosis of peripheral nerve entrapments or neuroma may be suspected by clinical history and physical examination and confirmed by EMG (the gold standard for nerve assessment) [8]. However, there are cases in which the evaluation by electroneurography and EMG are difficult, as in small nerves or terminal branches (such as the saphenous infrapatellar nerve, the Baxter nerve, and the medial and lateral plantar nerves). Therefore, the rapidly accumulating literature reinforces the use of US for diagnostic purposes, since high-resolution US provides an increasing amount of complementary morphological information about nerves and their surrounding tissues (loss of a normal fascicular pattern, thickening and hypoechogenicity, associated perineural fat stranding, and increased perineural vascularity) [9-11, 17-18], as observed in this case. Additionally, Tinel's sign may also be present in this location [11, 17].

The literature also refers to the importance and great accuracy of MRI diagnosis; however, given the greater accessibility and increasing accuracy (greater sensitivity and same specificity), it is possible that, in the future, US will become the gold standard for diagnosing peripheral neuropathies [10, 17-19]. It is currently agreed that US peripheral nerve screening by experienced physicians can be used for the diagnosis of nerve entrapment syndromes [10, 17-19]. In US, the disadvantage of interobserver variability must be taken into account [19].

As in this case, US has an additional role for guidance of nerve block that is diagnostic, as well as for guidance of other minimally invasive procedures, such as the ones performed (corticoanesthetic block, perineural hydrodissection, and radiofrequency) procedures that are gaining more evidence [8-9, 11, 17].

Conservative treatment should be tried before carrying out the described minimally invasive approaches, as happened in this clinical case, where the athlete was previously submitted to physiotherapy, correction of biomechanical defects, and pharmacological treatment with NSAIDs, analgesics, muscle relaxants, and pregabalin, as indicated in the literature [1-2, 6, 12-13], but without benefit.

As there was a recurrence of pain complaints, about three weeks after performing the corticoanesthetic blockade associated with perineural hydrodissection of the SPN, we moved on to effective and longer-lasting treatment, which can be attained using RF. This modality should be considered in refractory patients. Continuous and pRF, through nerve ablation or neuromodulation, respectively, can play an important role in the treatment of pain caused by these neuropathies [15, 16].

Although pRF mechanisms are still unclear, it is thought that pRF generates an electromagnetic field that has a neuromodulatory effect, interrupting gene expression in sensory neurons and sensitization-related molecules (pro-inflammatory cytokines tumor necrosis factor-α and interleukin-6, ion channels modulators, and neurotransmitters), activating descending noradrenergic and inhibitory serotonergic pathways, and inhibiting excitatory nociceptive C fibers, with special selectivity for C and Aδ fibers, without damaging of the surrounding tissue [15-16, 20]. With pRF, a low-energy electrical field in rapid pulses is generated, so tissue temperature remains at or below 42ºC, and no structural damage is caused, so there are fewer complications and side effects. Thus, this is the RF modality of choice for the treatment of neuropathies of mixed nerves (containing both motor and sensory fibers), SPN applies for both cases, but also mixed nerves can also have cutaneous representation, such as the SPN (sensory changes risk with thermal ablation of continuous RF) [15-16, 20]. So, with the pRF technique, we are left with the normal and elementary risks of all minimally invasive techniques [3, 9, 11-13].

pRF has shown positive results in relieving pain related to various neuralgias, including SPN [2-3]. In this case report, it can be observed a very quick relief of pain (two days after pRF treatment) that is maintained during an important period (two years), which becomes very relevant in elite athletes such as the one described.

As always in medicine, an adequate clinical exploration and an accurate diagnosis will be decisive for therapeutic success. As in this case, the high clinical suspicion, associated with a dynamic US exploration, which showed the alterations described compatible with neuropathy, associated with an immediate diagnostic procedure (anesthetic nerve block) and subsequent corticoanesthetic nerve block with hydrodissection (which allowed control of symptoms for about one month), was decisive for the final therapeutic success of pRF.

## Conclusions

This case report illustrates the efficacy of pRF in the treatment of SPN entrapment neuropathy, leading to pain resolution without any side effects or recurrence over two years. It allowed for an early return to play and helped avoid more invasive treatment options. An accurate diagnosis was crucial for successful treatment; therefore, peripheral neuropathies should be considered, particularly when there are neuropathic symptoms, to prevent underdiagnosis. The use of US can assist in the diagnosis, further enhancing the precision of the PRF technique.
